# Quantification of Antimicrobial Use on Commercial Broiler Farms in Pakistan

**DOI:** 10.3390/ani14233510

**Published:** 2024-12-04

**Authors:** Qamer Mahmood, Ilias Chantziaras, Jeroen Dewulf

**Affiliations:** Veterinary Epidemiology Unit, Department of Internal Medicine, Reproduction and Population Medicine, Faculty of Veterinary Medicine, Ghent University, Salisburylaan 133, 9820 Merelbeke, Belgium; ilias.chantziaras@ugent.be

**Keywords:** antibiotics, antimicrobial quantification, AMU metrics, defined daily dose, defined course dose, poultry, broilers

## Abstract

Pakistan has a large, intensive broiler production industry, where antimicrobials are extensively used for both therapeutic and prophylactic purposes. Monitoring antimicrobial use (AMU) at the farm level is crucial to guide interventions for antimicrobial stewardship. The objective of this study was to comprehensively quantify AMU on commercial broiler farms in Pakistan using different metrics. Data on on-farm AMU (both therapeutic and prophylactic) were collected from intensive commercial broiler farms in Punjab, Pakistan. AMU was quantified using four different metrics: treatment incidence (TI) based on defined daily dose (TIDDD_vetPK_), TI based on defined course dose (TIDCD_vetPK_), TI based on used daily dose (TIUDD_vetPK_), and mg/kg biomass. The median TIDDD_vetPK_, TIDCD_vetPK_, TIUDD_vetPK_, and mg/kg biomass values were 57.7 (7.5–257.9), 13.3 (1.8–52.5), 75.3 (21.1–182.9), and 301 (46.8–1009.6), respectively, based on 741 group treatments given at 100 participating farms with 225 flocks. About 34% treatments were administered for therapeutic purposes, with 66% administered as prophylaxis. A total of 41 active substances, distributed across 17 antimicrobial classes, were identified from 139 commercial antimicrobial products used. The most commonly used active substances (AS), out of total 741 group treatments, were colistin (polymyxins) at 17%, enrofloxacin (quinolones) at 8%, neomycin (aminoglycosides) and amoxicillin (aminopenicillins) at 7% each, and procaine penicillin (NS penicillins) and streptomycin (aminoglycosides) at 6% each. According to the WHO categorization, 57% of the used AS were found to be critically important antimicrobials (CIAs) for human medicine. As many as 30% of treatments were administered in the first week (13% on the first day of production), and the main indication was prophylaxis for early chick mortality, followed by 21% in week 5, with main indication being feed supplements for necrotic enteritis. The average weight at slaughtering was 2.25 kg, gained after an average of 44 days of the production cycle. The alarmingly high AMU in Pakistani broiler production emphasizes the urgent need to reduce AMU, particularly involving CIAs and the prophylactic use of antimicrobials. This can be achieved by improving farm management practices and strengthening regulatory oversight to enable prudent antimicrobial use, eventually reducing the risk of AMR.

## Simple Summary

Antimicrobials are extensively used in Pakistan’s broiler farming industry. However, their improper use can contribute to the development of antimicrobial resistance, posing a significant threat to both human and animal health. This study quantified antimicrobial usage at 100 broiler farms in Pakistan using four different quantification methods. The findings revealed that most treatments were administered to prevent diseases rather than to treat them, with many involving antimicrobials critical to human health. A total of 41 antimicrobial substances from 17 classes were identified, with colistin, enrofloxacin, and neomycin being the most commonly used. The majority of treatments were given during the first week of production. This study underscores the alarmingly high levels of antimicrobial use on these farms, emphasizing the need for urgent reductions, particularly in the use of antimicrobials classified as critically important for humans. Improving farm management practices and regulatory oversight can promote responsible antimicrobial use and help mitigate the risk of antimicrobial resistance, ultimately benefiting both human and animal health.

## 1. Introduction

Antimicrobials have revolutionized modern medicine by enabling effective treatments for many life-threatening diseases and saving countless lives. However, the emergence and spread of antimicrobial resistance (AMR) among pathogens has become a global health issue affecting humans, animals, and environment [1]. Recognizing AMR as a critical challenge, the World Health Organization (WHO) has emphasized the urgent need for concerted efforts to combat this growing threat [2].

Although evolved through natural processes, the development and spread of AMR is greatly influenced by misuse of antimicrobials in human and veterinary medicine [3,4]. This concern is particularly intensified in many low- and middle-income countries (LMICs), where antimicrobials are extensively used—mostly without a definite diagnosis or prescription—for treatment, prevention, and as growth promotors in livestock production [5,6].

In Pakistan, the livestock industry (including poultry), stands as the largest segment within agriculture, contributing 60.85% to the total agricultural output and 14.63% to the national GDP [7]. The main animal species used as food sources are poultry, cattle, and small ruminants. The poultry industry, especially broiler production, plays a crucial role in fulfilling the nutritional requirements of the population and driving economic growth. With 1703.36 million broilers constituting 32.7% of the total meat production annually, Pakistan ranks as the 11th largest poultry producer globally [8].

To meet the growing protein needs of 241.5 million human population, approximately 75–80% of broiler production in Pakistan consists of environmentally controlled intensive systems, with 70% based in the province of Punjab [8]. This intensive production requires the frequent administration of mass medication, leading to regular exposure of animals to antimicrobials. Due to the lack of any structural oversight, antimicrobials can be purchased and used without any prescription [9,10]. Antimicrobials are typically administered in group treatments, not only for treatment but also for prevention and growth promotion, often involving the use of Critically Important Antimicrobials (CIAs) for human medicine [11]. To tackle this imprudent use of antimicrobials, understanding the trends and quantifying volumes of antimicrobial use (AMU) in Pakistani broilers is imperative.

Many high-income countries have implemented farm-level AMU monitoring systems, which have been effective in reducing AMU in animals [12,13]. In contrast, LMICs, such as Pakistan, often lack national AMU monitoring systems, reflecting significant regional disparities in AMU regulation and practices [5,14]. Establishing such a system involves numerous challenges, particularly in selecting appropriate metrics for quantifying and reporting AMU. The metric serves as a technical unit to quantify an animal’s exposure to antimicrobials, influencing the interpretation of AMU results [15]. An increasing variety of AMU metrics is available in veterinary medicine, including treatment incidence (TI) based on defined daily dose (TIDDD_vetPK_), TI based on defined course dose (TIDCD_vetPK_), TI based on used saily dose (TIUDD_vetPK_), and mg/kg biomass, among others [16,17]. The suffix “vetPK” in metrics indicates veterinary metrics specific to Pakistan, where “vet” refers to veterinary antimicrobials and “PK” stands for Pakistan.

Depending on the objective and context of the study, two types of data can be collected for estimating AMU: population-level data, such as antimicrobial sales figures or import data; and practice-level data, which focuses on on-farm usage. In contrast to antimicrobial sales data, use-data at the farm and flock level can be more informative, as they consider the dosing variations among different species and antimicrobials [18].

To optimize the use of antimicrobials in Pakistani broiler production and ultimately reduce the risk of AMR development, quantitative monitoring of AMU is required at the farm and flock level [19]. This study aimed to quantify AMU on commercial broiler farms in Pakistan to identify the volumes and trends of antimicrobial usage, which can inform targeted interventions to reduce AMU and guide antimicrobial stewardship.

## 2. Materials and Methods

### 2.1. Study Design and Data Collection

**Figure 1:**
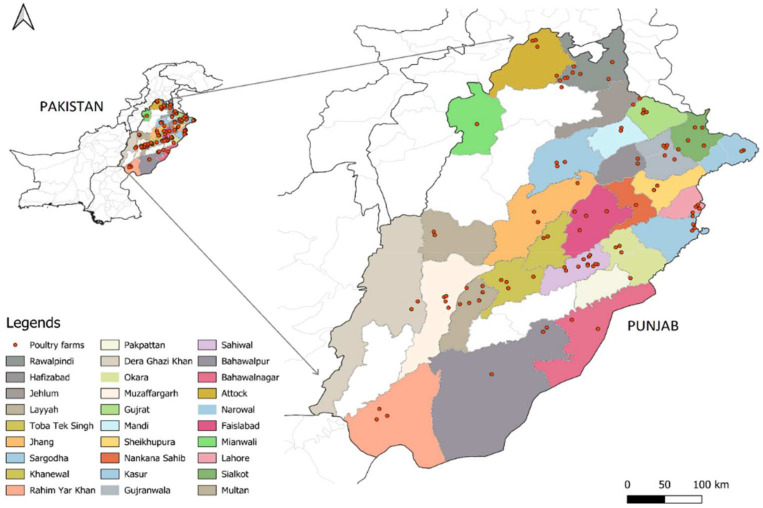
Data collection sites from 29 districts of Punjab province. The legend lists the names of each district, while the red dots indicate poultry farms within each district where data were collected. To protect the exact locations of the farms, GPS coordinates were anonymized.

In a cross-sectional survey, data on the use of antimicrobial active substances for treatment and prevention were collected through a farm visit and questionnaire. Farms were selected based on inclusion criteria to have environmentally controlled houses, a minimum of 20,000 birds per flock, an all–in/all-out production system, and a maximum rearing period of 35–50 days. Farmers were recruited on a volunteer basis, but a regional stratification was incorporated covering nearly all regions of Punjab (Figure 1), with a veterinarian (farm consultant) always accompanying during visits.

On-farm antimicrobial use data were collected with several parameters, including product name, number of active substances (AS), concentration of AS, actual used dose of the product, age (days) and weight (kg) of birds at treatment, duration of treatment and production cycle, daily feed and water intake, and number of birds at treatment and per round, among others. The full questionnaire is available in Supplementary Materials.

The antimicrobial products used during each production cycle, excluding antivirals, anticoccidials, and zinc oxide, were recorded from the farm’s daily treatment register. In this study, the term “antimicrobial” refers to any product with an antimicrobial mode of action that has been assigned an ATCvet code by the WHO Collaborating Centre [20]. To address the global concern of AMR, the WHO has categorized medically important antimicrobials based on their potential risk of developing and spreading AMR and the risk management associated with AMU in non-human sectors. These categories are as follows: highest priority critically important antimicrobials (HPCIA), critically important antimicrobials (CIA), highly important antimicrobials (HIA), and important antimicrobials (IA) [21]. The antimicrobials reported in this study were categorized according to this scheme.

Data on antimicrobials used for therapeutic or prophylactic purposes were collected through a questionnaire, while no detailed information was available regarding the administration of antimicrobial growth promoters (AGPs) in the treatment register. For each antimicrobial administered, the purpose (whether therapeutic or prophylactic) was obtained directly from the farm’s record register. In cases where the purpose was not clearly stated in the records, the farm veterinarian was consulted to ensure accurate completion of the information. Data on AGPs were collected from farm veterinarians and feed mill officers, as AGPs are typically mixed into poultry feed at the feed mill level, and farmers are often unaware of the actual concentration in the feed. The definitions of the terminologies used for antimicrobials in this study were adapted to align with the specific practices and context of Pakistan (Table 1) [22].

### 2.2. AMU Quantification

AMU (both therapeutic and prophylactic) was quantified using four different metrics. AGPs were not quantified as no detailed information was available by farmers on the specific dosage, indication, and duration of AGPs administered during the production cycle. In the calculations, each AS was considered a single treatment [23], except for synergistic combinations, where a combination of two AS was regarded as a single treatment.

#### 2.2.1. TI Based on DDD_vetPK_

TI was calculated for each treatment using the total amount of AS administered during the production cycle (in milligrams) as the numerator. The denominator consisted of the DDD_vetPK_ value for that specific AS, along with the total number of days at risk (rearing period) and the kilograms of animal at risk (kg AAR) [18].

$${\mathrm{TIDDD}}_{\mathrm{vetPK}}=\frac{Total\;amount\;of\;active\;substance\;administered\;(mg)}{DDDvetPK\;((mg/kg)/day)\times No.of\;days\;at\;risk\times kg\;AAR}\;\times \;100\;\mathrm{AAR}$$

The numerator was calculated using following formula [18]:

Total amount of active substance administered (mg) = UDD (mg/kg/day) × treatment duration (days) × no. of animals treated × actual weight at treatment per animal (kg).

Here, TI is expressed as the number of DDD_vetPK_ administered per 100 animal-days at risk. Simply put, it reflects the percentage of time that a broiler is treated with antimicrobials in its life. DDD_vetPK_ reflects the average recommended dose of an AS per kg of broiler per day [18]. For antimicrobials used in broiler production in Pakistan, DDD_vetPK_ and DCD_vetPK_ values were recently established based on the recommended dose and duration of an active substance specified in the Summary of Product Characteristics (SmPC) [24]. “Kg AAR” was calculated by multiplying the number of broilers treated with actual weight at treatment. “Number of days at risk” was assessed by taking the average of various slaughtering group durations per production cycle at each farm. UDD represents the actual dose of AS (in mg/kg) administered at the farm. TIDDD_vetPK_ at the farm level was calculated by adding up the TIs of all treatments at each farm.

#### 2.2.2. TI Based on DCD_vetPK_

TI based on DCD_vetPK_ was calculated in the same manner as for TIDDD_vetPK_, except that the DCD_vetPK_ value for each specific AS was used in place of the DDD_vetPK_ value in the denominator.

$${\mathrm{TIDCD}}_{\mathrm{vetPK}}=\frac{Total\;amount\;of\;active\;substance\;administered\;(mg)}{DCDvetPK((mg/kg)/day)\times No.of\;days\;at\;risk\times kg\;AAR}\;\times \;100\;\mathrm{AAR}$$

TIDCD_vetPK_ expresses the number of treatment courses per 100 animal-days at risk, where DCD_vetPK_ reflects the average recommended dose of a drug per kg of broiler per treatment course [18]. Just like TIDDD_vetPK_, TIDCD_vetPK_ at the farm level was calculated by adding up the TIs of all treatments at each farm.

#### 2.2.3. TI Based on UDD_vetPK_

When based on UDD_vetPK_, TI becomes the number of days broilers underwent antimicrobial treatment normalized by total days of production cycle.

$${\mathrm{TIUDD}}_{\mathrm{vetPK}}=\frac{Number\;of\;days\;with\;antimicrobial\;treatment}{Total\;number\;of\;days\;at\;risk}\;\times \;100\;\mathrm{AAR}$$

TI based on UDD_vetPK_ simply counts the number of days the broilers were treated with a certain AS during the production cycle irrespective of the dose used. Multiplying the formula with 100 AAR results in a number of UDD_vetPK_ per 100 broilers at risk per day. Total TIUDD_vetPK_ at all farms was calculated by adding up the TIUDD_vetPK_ values of all treatments at each farm.

#### 2.2.4. Milligram/Kilogram Biomass

This metric has the same numerator as TI based on DDD_vetPK_ and DCD_vetPK_ but a different denominator. Instead of using the dose of AS, it takes into account the weight of animals at treatment [25].

$$\mathrm{Milligram}/\mathrm{kg}\;\mathrm{biomass}=\frac{Total\;amount\;of\;active\;substance\;administered\;\left(mg\right)}{Actual\;body\;weight\;per\;animal\;at\;treament\times No.of\;animals\;at\;treatment}$$

The values of mg/kg biomass at the farm level were calculated by adding up the mg/kg biomass values of all treatments at each farm.

### 2.3. Data Analysis

Descriptive statistics were performed in Microsoft Excel. All traceable data of farms were anonymized. To show the distribution of sampled farms in the study population, a QGIS map was produced using GPS coordinates. A scatter plot and Spearman’s rank correlation test were conducted in R to evaluate the correlation between various AMU metrics. To visualize the relative difference in AMU estimation at the farm level based on different metrics, a grouped bar plot was constructed using the ggplot2 package in R. The proportions of each antimicrobial class were calculated for four metrics: TIDDD_vetPK_, TIDCD_vetPK_, TIUDD_vetPK_, and mg/kg biomass. To calculate and report total AMU per farm, the median was considered to be the most informative measure rather than the arithmetic mean given the skewed distribution of data.

## 3. Results

### 3.1. Farm Characteristics

Out of 100 sampled farms in this study, the number of flocks per farm ranged between 1 to 10 with a maximum number of broilers at risk, ranging from 20,000 to 236,936 per farm. In total, 255 flocks were included representing 6.84 million broilers. All data were collected between January and April 2023. The average weight of day-old chicks (DOC) was 40 g. At the time of farm visits, chicks were, on average, 31 days old, weighing approximately 1.5 kg. Each farm averaged six production cycles per year. The average slaughter weight was 2.25 kg, with a production cycle lasting 44 days. On average, each farm used 1,723,625.65 kg of feed annually, and 380,303 birds were sent for slaughter per year.

### 3.2. AMU at the Farm Level

**Figure 2:**
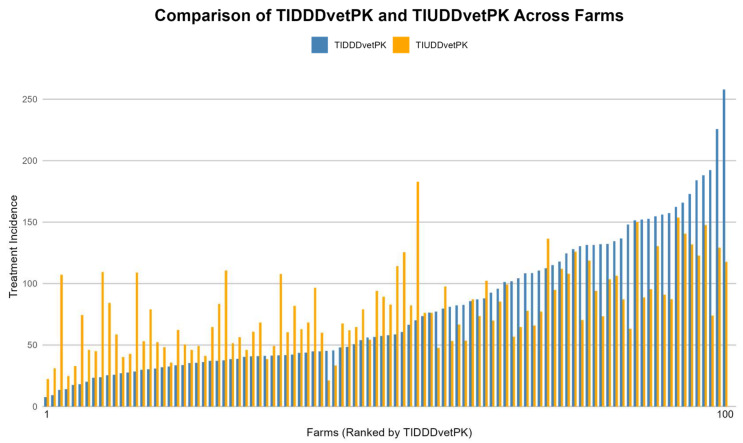
Comparison of treatment incidence based on defined daily doses (DDD_vetPK_) and used daily doses (UDD_vetPK_) across 100 broiler farms. The farms are ranked from lowest to highest based on TIDDD_vetPK_ to highlight differences between theoretical and actual antimicrobial use. Each bar on the *x*-axis represents antimicrobial usage at a sampled farm.

All 100 surveyed farms used antimicrobials, and all treatments were administered as group treatment, with no individual treatments reported. Based on 741 group treatments given at all farms, the median TIDDD_vetPK_, TIDCD_vetPK_, TIUDD_vetPK_, and mg/kg biomass were 57.7 (7.5–257.9), 13.3 (1.8–52.5), 75.3 (21.1–182.9), and 301 (46.8–1009.6), respectively. In other words, broilers were treated with antimicrobials (either for preventive and/or therapeutic purpose) during almost 58% (based on TIDDD_vetPK_) or 75.3% (based on TIUDD_vetPK_) of their lifespan, with an average of 13 antimicrobial courses per 100 days of production, which is around 6 to 7 courses per production cycle of 44 days. Depending on the metric used to quantify AMU, the outcome may vary accordingly, as shown in Figure 2, where TI based on DDD_vetPK_ and UDD_vetPK_ was calculated across all farms. Out of 139 commercial antimicrobial products, 55% had more than one AS (2–4). About 77% of treatments were administered through drinking water, while the rest (23%) were administered through feed.

### 3.3. AMU per Antimicrobial Class

In total, 139 antimicrobial products were used at all sampled farms. These products contained 41 different AS belonging to 17 antimicrobial classes. Each AS was considered to be a separate treatment except for AS combinations showing synergistic effects. This includes lincomycin and spectinomycin combinations, and sulfonamides combinations with trimethoprim. The most commonly used AS out of total 741 group treatments were colistin (polymyxins) at 17%, enrofloxacin (quinolones) at 8%, neomycin (aminoglycosides) and amoxicillin (aminopenicillins) at 7%, and procaine penicillin (narrow spectrum penicillins) and streptomycin (aminoglycosides) at 6% (Table 2). In our dataset, 57% of AS were found to be critically important for human medicine, with 30% HPCIA and 27% CIA.

**Figure 3:**
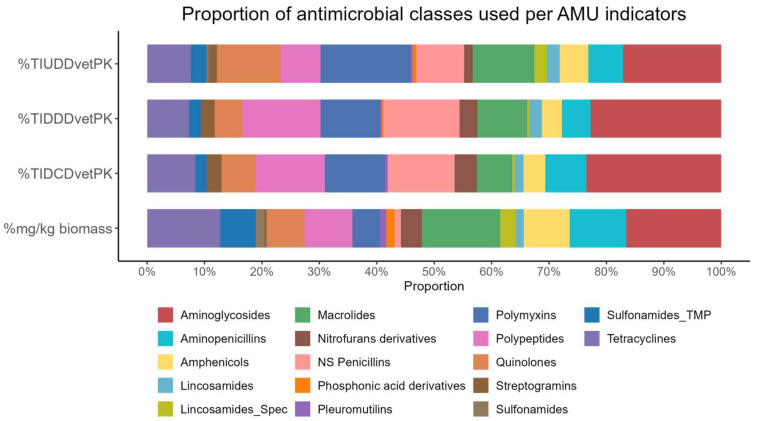
Proportional use of antimicrobial classes across four AMU metrics at the farm level. The metrics shown are %TIUDD_vetPK_, which represents the proportion of antimicrobial usage based on the used daily dose per kg of animal; %TIDDD_vetPK_, representing the proportion based on the defined daily dose in mg/kg; %TIDCD_vetPK_, which is the proportion based on the defined course dose in mg/kg; and %mg/kg biomass, showing the proportion of antimicrobial usage normalized by milligrams per kilogram animal biomass. Each bar is stacked to show the relative contribution of different antimicrobial classes to overall usage at all farms for each metric, and the proportions are displayed as percentages, summing to 100% for each metric.

Figure 3 highlights the proportional use of 17 antimicrobial classes as described by four different AMU metrics.

### 3.4. AMU per Indication

**Figure 4:**
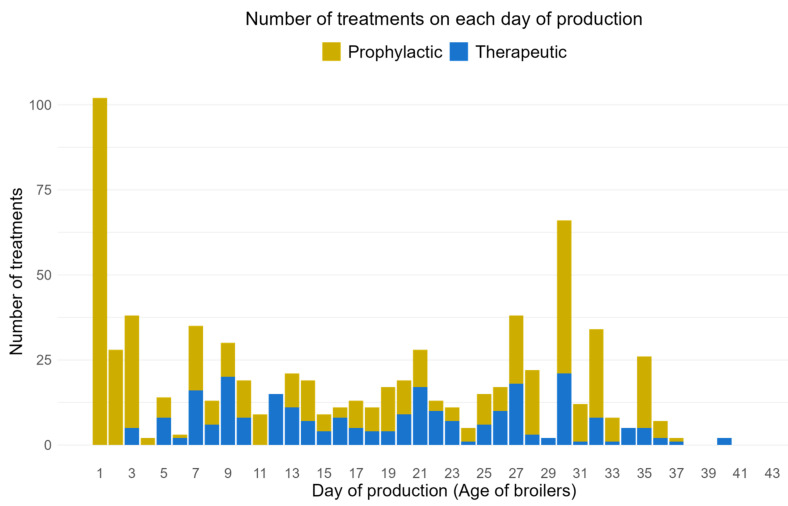
A grouped bar plot showing the number of antimicrobial treatments administered to broilers on each day of production. No treatments were administered on day 38, 39, 41, 42, 43, and 44 of production day.

Out of the total 741 group treatments, 34% AS were administered for therapeutic purposes, while 66% were used as prophylaxis (Figure 4). Indication varied depending on the age of the broilers. A total of 102 treatments were administered on day one and all were prophylactic. This was the highest number of treatments in a single day of production. The most common indication on the first day was “early chick mortality” (70%), followed by “chronic respiratory disease” (7%), colibacillosis, and enteritis (4% each). Over the first week, the “early chick mortality” was again the most frequently used reason to administer antimicrobials, accounting for 46% of treatments out of 222 total treatments (both therapeutic and prophylaxis) during the whole first week. During the second and third week of age, chronic respiratory disease was the most common indication, with 18% (out of 126) and 16% (out of 108) treatments, respectively. Feed supplements for necrotic enteritis and chronic respiratory disease were most frequently reported during the fourth and fifth week of production cycle with 33% (out of 121) and 52% (out of 153) treatments, respectively. Furthermore, the type of antimicrobial used varied widely throughout the rearing period. Colistin was given at least once during the 33 days of production cycle out of 44 days, followed by doxycycline (25 days) and lincomycin (23 days).

Table 3 shows the distribution of antimicrobial use for both therapeutic and prophylactic purposes in broilers across various diseases and conditions.

### 3.5. AMU per Production Cycle

**Figure 5:**
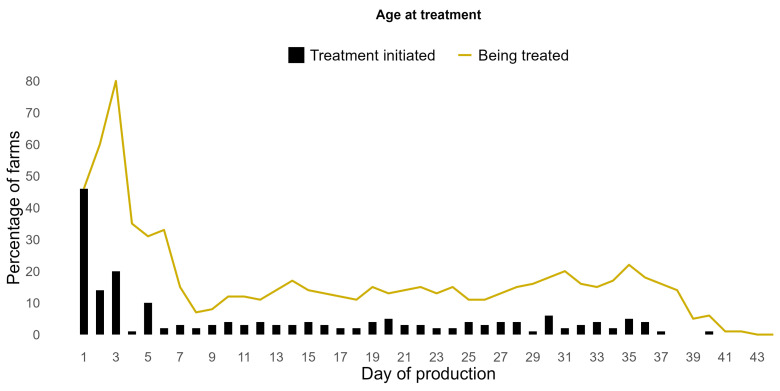
The histogram shows the proportion (%) of farms (*y*-axis) where treatment with antimicrobials was initiated on a certain age (days) (*x*-axis). The line shows the proportion (%) of farms that were being treated with antimicrobials on a certain day of production. The *x*-axis represents the age of broilers in days; it covers one rearing period from day 1 until slaughter.

At 46% of the farms, antimicrobial treatments were initiated on the first day of production, followed by 14% and 20% on days 2 and 3, respectively (Figure 5). As a result, 80% of farms were being treated with antimicrobials on day 3. Subsequently, after the first week, the percentage of farms where antimicrobials were being administered gradually decreased to 2% on day 8, followed by a slight increase resulting in a fluctuation between 10–20% during the second, third, and fourth weeks. A slight increase in treatments was observed again during the fifth week as many farms initiated feed supplements at the end of fourth week and continued it during fifth week. No new treatments were initiated from day 37 onwards, except for one on day 38.

### 3.6. Comparison of UDD_vetPK_ and DDD_vetPK_

The UDD_vetPK_/DDD_vetPK_ ratios were calculated for each AS, separately for treatment and preventive indications. A value of 1 indicates the same dose for UDD_vetPK_ and DDD_vetPK_, while values less or more than 1 indicate underdosing and overdosing, respectively (Table S1).

Among AS used for treatment, the UDD/DDD_T_ ratios varied considerably, ranging from 0.2 to 9.0. The mean UDD/DDD_T_ ratio was 1.1, indicating that, on average, the actual doses used for treatment were close to the DDDs. AS such as sulfadiazine (3.1) and trimethoprim_sulfa had notably higher ratios (9.0), suggesting that these were used at doses greater than their DDDs. In contrast, AS like norfloxacin (0.4) and trimethoprim (0.2) were used at doses lower than their DDDs.

For AS used for preventive purposes, the UDD/DDD_P_ ratios ranged from 0.2 to 2.9, with a mean ratio of 1.2. This suggests that the actual doses used for preventive purposes were generally close to or slightly higher than the DDDs. Some exceptions include furaltadone, which had a high ratio of 2.9, indicating a considerably higher actual dose compared to the DDD, and norfloxacin, which had a very low ratio of 0.2, implying that its preventive use was much lower than the DDD.

### 3.7. Relationship Between AMU Metrics

**Figure 6:**
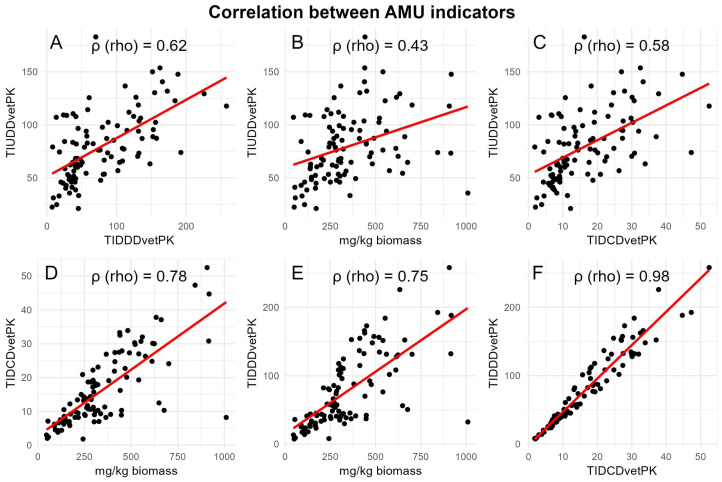
Scatter plots showing the correlation between four AMU metrics based on AMU data per farm. “p (rho)” represents value of correlation coefficient for each pair of metrics. The black dots in each plot represent total AMU par farm, while the red line indicates the linear regression fit showing a positive or negative relationship between two metrics. (**A**) Correlation between TIUDD_vetPK_ and TIDDD_vetPK_; (**B**) correlation between TIUDD_vetPK_ and mg/kg biomass; (**C**) correlation between TIUDD_vetPK_ and TIDCD_vetPK_; (**D**) correlation between TIDCD_vetPK_ and mg/kg biomass; (**E**) correlation between TIDDD_vetPK_ and mg/kg biomass; (**F**) correlation between TIDDD_vetPK_ and TIDCD_vetPK_.

To check the relationship between AMU metrics, a linear regression model along with a spearman’s correlation coefficient p (rho) was calculated (Figure 6). TIDDD_vetPK_ and TIDCD_vetPK_ exhibited the strongest correlation (rho = 0.98), indicating that both metrics capture almost similar aspects of antimicrobial usage. While TIUDD_vetPK_ and mg/kg biomass showed the weakest correlation (rho = 0.43).

## 4. Discussion

In the present study, antimicrobial use in intensive commercial broiler farms in Pakistan was described and quantified using four different AMU metrics. Previously, only a few studies on AMU quantification exist in Pakistan that use one metric (mg/kg biomass) [26,27] or the European Medicine Agency’s DDD_vet_ values [10,28] or antimicrobials import data [11]. In contrast to many high-income countries, a formal surveillance of AMU in food animals is absent in Pakistan, largely due to factors such as weak legislation, poor enforcement of existing laws, and limited resources, among other factors [6,10,28]. Moreover, the absence of any standardized AMU metrics, as compared to international standards, presents a difficulty in comparing the data across farms, species, production type, and countries [29]. To our knowledge, this is the first study in Pakistan to quantify AMU comprehensively, across 100 farms, using locally determined TIDDD_vetPK_, TIDCD_vetPK_, and TIUDD_vetPK_ metrics in addition to mg/kg biomass. Collecting data at the farm level is important for understanding the reasons behind and methods of large-scale antimicrobial usage. It also helps to identify high users and can lay the groundwork for developing a systematic antimicrobial stewardship program [23]. While a farm veterinarian was always present during the visits to supplement any missing information in the farm treatment registers, self-reported data could still have limitations and might not fully capture all treatments administered on the farm.

The median TIDDD_vetPK_ value of 57.7 at farm level, calculated in the present study, was much higher as compared to the TIDDD_vet_ value for many European countries, such as Belgium (12.5), Bulgaria (15.5), Denmark (0.0), France (50.3), Germany (1.5), Italy (4.0), Poland (17.8), Spain (10.8), and the Netherlands (8.5) [18]. Our results were, however, comparable to the TIDDDvet of 60, recently calculated for Bangladesh [30]. Similarly, the median TIDCDvetPK value of 13.3 was higher than that of many European countries: Belgium (2.7), Bulgaria (3.2), Denmark (0.0), France (9.8), Germany (0.3), Italy (0.9), Poland (3.9), Spain (2.4), and The Netherlands (2.1) [18]. Unlike TIDDD_vetPK_, TIDCD_vetPK_ was slightly higher than TIDCD_vet_ value for Bangladesh (11.9) [30]. The median mg/kg biomass value for all farms was 301 in this study, which is lower than another study conducted in Pakistan showing 462.57 mg/kg biomass [28] based on 19 farms. TIUDD_vetPK_ in this study ranged from 21.1 to 182.9. This is due to the fact that we considered each AS as a single treatment, so in the case of products having 3–4 AS within one product, with a duration of 12 days for each for instance, this would result in more treatment days than the production cycle. In reality, these AS were given in parallel on the same days at farm.

The higher antimicrobial usage in Pakistan’s broiler production compared to other countries can be attributed to factors such as the low biosecurity levels and the lack of a comprehensive regulatory framework with which to govern antimicrobial use [6,27,31]. Economic constraints may also play a role as farmers often rely on antimicrobials to mitigate potential losses due to diseases that could impact their productivity and profitability [32]. The broiler production in Pakistan primarily functions as a live bird market due to the lack of cold storage facilities and the consumer preference for fresh chicken [33]. This means that farmers are under constant pressure to quickly bring birds to market as they must be sold fresh. This market structure, coupled with price volatility, can lead to higher antimicrobial use as farmers may rely on antibiotics to reduce disease risk and ensure rapid growth during short production cycles. In small-scale farms (20–30 thousand birds per flock), veterinarians are often not consulted regularly. Instead, representatives from pharmaceutical companies establish direct contact with farmers, providing them with antimicrobials and offering consultancy services. These services, however, are often profit-driven rather than focused on animal health or best practices. Additionally, there is no widespread use of proper laboratory diagnostics to guide the use of antimicrobials. The concept of drug withdrawal periods and testing the efficacy of drugs is poorly understood by farmers [31]. Another important issue is that pharmaceutical companies provide antimicrobial doses for administration via feed and water in the SmPC. The drug regulatory authority should standardize the SmPC by mandating dosage instructions in mg/kg body weight rather than per liter of water or kilogram feed. This approach would reduce self-medication practices by farmers and ensure greater oversight by veterinarians, ultimately promoting responsible AMU.

AMU per production cycle varied considerably among farms, indicating differences in how antimicrobials are administered. This variation suggests that some farmers are already able to raise broilers with lower antimicrobial usage. Further research is needed to identify the practices that enable these farms to use fewer antimicrobials. These successful examples could be shared with higher-usage farms to demonstrate that broilers can be raised with far less AMU. Factors influencing these differences might include disease management strategies, varying biosecurity measures, or differences in awareness and compliance with guidelines [34,35,36].

The high proportion of CIAs found in our dataset raises concerns in the context of AMR risk management. The widespread use of fluoroquinolones and polymyxins, which are both on the HPCIA list by WHO [21], may be driven by their perceived efficacy in treating common infections in poultry. Other studies also showed similar trends [11,26,27,28].

AMU showed the highest peak on the first day of production, with almost 14% of all treatments (*n* = 741) administered at 46% (*n* = 100) of farms, all aimed at preventing early chick mortality for prophylactic purposes. Research indicates that stressors such as transportation, temperature fluctuations, and exposure to pathogens significantly compromise the immune systems of day-old chicks, making them highly susceptible to infections [37]. As the birds grew older, the focus shifted to CRD, which became the leading indication by the second and third weeks. This shift can be attributed to the intensive rearing conditions, characterized by high stocking density and suboptimal ventilation, which create a perfect environment for respiratory infections [38]. In the later weeks, there was an increased use of feed supplements aimed at managing necrotic enteritis, often triggered by changes in diet and the stresses of rapid growth, posing significant risks during this period. Farmers may choose to incorporate antimicrobials into feed as a preventive strategy to maintain gut health and reduce the incidence of enteric diseases, which can lead to poor feed conversion ratios and increased mortality [39]. This type of mass medication is considered a cheap and easy solution for disease prevention [18].

In Pakistan, similar to many other LMICs, a significant proportion of AMU in animals was attributed to prophylaxis. Additionally, AGPs were also reported to be routinely used in poultry feed [40]. These AGPs are normally added into poultry feed at feed mills. The following concentrations were reported: salinomycin 0.05%, enramycin 0.25%, lincomycin 0.10%, and avilamycin 0.10% per 50 kg feed bag. Similar findings were recently reported in another study from Pakistan [6]. We have deliberately chosen not to include the AGP’s use in the quantification of the AMU as continuous use of AGPs throughout the production cycle automatically results in a 100% treatment incidence. Moreover, AGPs lack specific dosage, indication, and duration of usage, making it virtually impossible to estimate their actual on-farm usage. While the Punjab province has introduced a list of permitted AGPs [41], there is still limited enforcement and oversight at the national level, allowing for widespread, unchecked use of AGPs [6]. Missing information about the exact concentrations of AGPs can certainly underestimate the actual antimicrobial usage. Collecting feed samples from various feed mills and conducting pharmacological analyses would help determine the precise concentrations of AGPs being used.

The selection of a metric to quantify AMU depends on several factors, including the objectives and context of the study, as well as the availability of data and resources. The choice of metric has great influence on the interpretation of AMU [17,23]. The TIUDD_vetPK_ and TIDDD_vetPK_ varied significantly among farms in our dataset. This variation highlights the issue of the underdosing and overdosing of used AS as compared to their recommended dose (DDD_vetPK_). A lower TIDDD_vetPK_ indicates that animals were often treated for more days than recommended, but with doses below the DDD_vetPK_, leading to a higher TIUDD_vetPK_ value. Conversely, a higher TIDDD_vetPK_ and lower TIUDD_vetPK_ suggest that these farms used higher doses than recommended but for fewer days.

To accurately describe and compare AMU at the farm or flock level, it is essential to use a metric with high spatial resolution (on-farm precision) and good comparability across farms [15]. We calculated four different metrics in this study. The “mg/kg biomass” metric does not take into account the differences in dosing and variation in the length of the production cycle, implying that kilograms of animals produced are exposed to antimicrobials over a full year. However, short-lived animals, such as broilers, are only at risk of antimicrobial exposure during their much shorter lifespan of around 1 month, not for the entire year. This miscalculation can lead to misinterpretations when comparing AMU across different species or countries. A key advantage of mg/kg biomass metric is that is easy to calculate compared to other methods, especially in resource-limited settings like Pakistan. Also, it is a useful metric for providing rough estimates and monitoring AMU at the population level. TIUDD_vetPK_ is easy to calculate and communicate as it is based on the number of treatment days normalized by the total days of production. It takes into account the time an animal is exposed to antimicrobials. This metric could be useful for research purposes, but it demands more detailed data. TIDDD_vetPK_ and TIDCD_vetPK_ provide more detailed data on on-farm AMU as they take into account the dosing difference among antimicrobials and various animal species, along with considering the time an animal is exposed to antimicrobials in its lifespan. These metrics can be a good choice for the surveillance and benchmarking of farms/farmers as they are based on practice level data. However, they demand more time and resources, as well as more quality data, and the calculation can sometimes be misinterpreted as the defined daily and course doses does not reflect the actual usage at farms. Another challenge could be the choice of dosage and animal weight to be used in calculations.

An important factor in the calculation of all these metrics is the weight of broilers used in the denominator. In Pakistan, the weight increases generally by a factor of nearly 50 (from 40 g day old chick to on an average of 2200 g final weight). If a single standard weight (normally 1 kg) is used, this could cause an under or over estimation of AMU given the fact that many treatments are given either in the first week of production or a week before slaughtering. We decided to use the actual weight of animals at treatment as this is closer to the reality and can provide more realistic insights into AMU at the farm level. This, however, can limit the comparability of our data with those of other countries.

## 5. Conclusions

The findings of this study highlight the alarmingly high use of antimicrobials in broiler production in Pakistan. Immediate action is needed to reduce AMU, particularly concerning the use of CIAs and growth promoters, while also restricting prophylactic and therapeutic use. The findings of this study should stimulate further analysis and a sense of urgency in accurately estimating and reducing the burden of AMU in broiler production in Pakistan and other neighboring countries, which have similar animal husbandry practices. This should ultimately lower the selection pressure for AMR.

## Figures and Tables

**Table 1:** Definitions of terms used for antimicrobials in this study.

Term	Definition
Therapeutic antimicrobial use	Administration of antimicrobials to treat animals with clinical evidence of a disease; e.g., amoxicillin, ampicillin, enrofloxacin.
Prophylaxis	Administration of antimicrobials to animals without evidence of clinical signs of a disease. The animals might be at high risk of infectious disease but there is no known disease in the flock (for example, conditions due to environmental change, crowded space, and transport of animals); e.g., bacitracin, benzylpenicillin, virginiamycin.
Antimicrobial growth promoters (AGPs)	Administration of sub-therapeutic doses of antimicrobials through feed to stimulate growth and weight gain in animals or to increase feed efficiency; e.g., enramycin, avilamycin, lincomycin.

**Table 2:** Percentage of the most used active substances out of total (*n* = 741) group treatments, stratified by antimicrobial class.

Antimicrobial Class (Active Substance)	Medical Importance	Percentage
Polymyxins (Colistin)	HPCIA	17%
Aminoglycosides (Neomycin, Streptomycin, Gentamicin, Apramycin, Dihydrostreptomycin)	CIA	15%
Quinolones (Enrofloxacin, Norfloxacin, Pefloxacine, Flumequine, Ofloxacin)	HPCIA	12%
Macrolides (Tylosin, Tilmicosin, Spiramycin, Erythromycin)	CIA	12%
Tetracyclines (Doxycycline, Chlortetracycline, Oxytetracycline)	HIA	9%
Aminopenicillins (Amoxicillin, Ampicillin)	HIA	7%
NS Penicillins (Procaine Penicillin)	HIA	6%
Amphenicols (Florfenicol, Chloramphenicol)	HIA	6%
Lincosamides (Lincomycin)	HIA	5%
Polypeptides (Bacitracin, Enramycin)	IA	5%
Sulfonamides (Sulfadiazine, Sulfamethoxypyridazine, Sulfadimerazine, Sulfamethoxine, Sulfachlorpyridazine, Sulfadiamidine, Sulfamethoxazole)	HIA	4%
Nitrofurans derivatives (Furaltadone)	IA	2%
Streptogramins (Virginiamycin)	HIA	1%
Phosphonic acid derivatives (Fosfomycin)	HPCIA	1%

HPCIA, highest priority critically important antimicrobials; CIA, critically important antimicrobials; HIA, highly important antimicrobials; IA, important antimicrobials.

**Table 3:** Percentage distribution of antimicrobial use across sampled farms, categorized by disease indication and purpose (prophylactic and therapeutic).

Category	Indication	Treatment (%, *n* = 252)	Prophylaxis (%, *n* = 489)	Total (%, *n* = 741)
Enteric Diseases	Feed supplement for necrotic enteritis	0	21.9	14.4
	Enteritis	4.4	4.7	4.6
	Necrotic enteritis	0.8	0.8	0.8
	Diarrhea	2	0.6	1.1
	Salmonellosis	6	1.2	7.2
	Shigellosis	0.8	0	0.3
Respiratory Diseases	Chronic respiratory disease (CRD)	24.2	9.2	14.3
	Feed supplement for CRD	0	10.2	6.7
	Respiratory distress	5.2	3.7	4.2
	Infectious coryza	5.6	1.4	2.8
	Infectious sinusitis	7.1	0	2.4
	Complex chronic respiratory disease	1.6	2.9	2.4
	Avian influenza	0	2.9	1.9
	Airsacculitis	1.6	1.6	1.6
	Pneumonia	0.4	0.6	0.5
	Avian coryza	0	0.4	0.3
Systematic Diseases	Colibacillosis	21	7.8	12.3
	Fowl cholera	4	2.5	3
	Acute fatal septicemia	0.4	1.2	0.9
	Colisepticemia	1.6	0.4	0.8
	Septicemia	1.6	0.4	0.8
	Pericarditis	1.2	0.4	0.7
	Peritonitis	0.4	0.6	0.5
General Diseases/Conditions	Early chick mortality	0	18.4	12.1
	Omphalitis	1.2	0.4	1.6
	Cellulitius	0	0.2	0.1
	Infectious sinuvitis	1.6	0.6	0.9
Locomotive Diseases	Arthritis	1.6	0.4	0.8
	Bumblefoot	0.4	0	0.1

The data shown are based on a total of 741 group treatments administered to broilers during rearing period from day 1 to 44.

## Data Availability

The raw data supporting the conclusions of this article will be made available by the authors on request.

## Supplementary Materials

The following supporting information can be downloaded at: https://www.mdpi.com/article/10.3390/ani14233510/s1, Table S1. Comparison of used daily dose (UDDvetPK) with defined daily dose (DDDvetPK) in mg/kg for all active substances used at surveyed farms; Questionnaire.
