# Supplementary material for: Quantification of Antimicrobial Use on Commercial Broiler Farms in Pakistan
Source: Animals (Basel). 2024 Dec 4;14(23):3510. doi: 10.3390/ani14233510 (PMC11640401; doi:10.3390/ani14233510)
Supplement: Supplementary file 1 [file animals-14-03510-s001.zip › animals-3306641-supplementary.pdf]

Supplementary materials

Table S1. Comparison of used daily dose (UDD_{vetPK}) with defined daily dose (DDD_{vetPK}) in mg/kg for all active substances used at surveyed farms.

Active substance	Antimicrobial class	Treatment (mg/kg)			Preventive (mg/kg)		
		UDD_{vetPK}	DDD_{vetPK}	UDD/DDD_T	UDD_{vetPK}	DDD_{vetPK}	UDD/DDD_P
Amoxicillin	Aminopenicillins	13.6	17.5	0.8	11	11.8	0.9
Ampicillin	Aminopenicillins	173.1	173.2	1.0	-	-	-
Apramycin	Aminoglycosides	62.9	99	0.6	-	-	-
Bacitracin	Polypeptides	-	-	-	13.8	8.1	1.7
Chloramphenicol	Amphenicols	6.6	10.6	0.6	-	-	-
Chlortetracycline	Tetracyclines	14	20.5	0.7	8.9	11.4	0.8
Colistin	Polymyxins	6.1	4.9	1.2	1.7	3.3	0.5
Dihydrostreptomycin	Aminoglycosides	1.4	2.6	0.5	-	1.3	-
Doxycycline	Tetracyclines	20.3	20.5	1.0	18.6	13.5	1.4
Enramycin	Polypeptides	-	-	-	1.1	0.8	1.4
Enrofloxacin	Fluoroquinolones	18.7	14.8	1.3	3.8	10.3	0.4
Erythromycin	Macrolides	9.6	12.7	0.8	4.9	7.4	0.7
Florfenicol	Amphenicols	19.5	22.7	0.9	13.2	13.9	0.9
Flumequine	Other quinolones	12.5	26.4	0.5	6.2	13.2	0.5
Fosfomycin	Phosphonic acid derivatives	22.6	28.3	0.8	-	13.2	-
Furaltadone	Nitrofurans derivatives	21.3	24.4	0.9	28.3	9.9	2.9
Gentamicin	Aminoglycosides	26.2	9.8	2.7	3.2	-	-
Lincomycin	Lincosamides	8.6	9.3	0.9	3.8	3.9	1.0
Lincomycin_spec[1]	Lincosamides	19.6	19.1	1.0	12.2	11.8	1.0
Neomycin	Aminoglycosides	18.7	13.3	1.4	9.3	6.4	1.5
Norfloxacin	Fluoroquinolones	11.6	28.2	0.4	2.2	9.7	0.2
Ofloxacin	Fluoroquinolones	17.3	19.8	0.9	8.3	-	-
Oxytetracycline	Tetracyclines	17.3	22.4	0.8	13.6	13.4	1.0
Pefloxacine	Fluoroquinolones	17.2	17.6	1.0	10.3	8.8	1.2
Procaine Penicillin	Natural Penicillins	-	-	-	1.3	1	1.3
Spectinomycin_linc[2]	Aminocyclitols	19.7	32.6	0.6	12	19.2	0.6
Spiramycin	Macrolides	4.3	8.9	0.5	7.6	4.9	1.6
Streptomycin	Aminoglycosides	-	-	-	5.5	4.1	1.3
Sulfachlorpyridazine_TMP[3]	Sulfonamides	11.3	20	0.6	-	-	-
Sulfadiazine_TMP[3]	Sulfonamides	75.4	24.1	3.1	13.7	8.8	1.6
Sulfadimerazine	Sulfonamides	67.4	113.5	0.6	17.5	-	-
Sulfadimidine_TMP[3]	Sulfonamides	-	52.8	-	31.4	26.4	1.2
Sulfamethoxazole_TMP[3]	Sulfonamides	16.3	37	0.4	-	-	-
Sulfamethoxine_TMP[3]	Sulfonamides	-	19.8	-	6	9.9	0.6

Sulfamethoxypyridazine_TMP[3]	Sulfonamides	11.2	20.9	0.5	9.3	10.5	0.9
Tiamulin	Pleuromutilins	21.8	57	0.4	34.3	33	1.0
Tilmicosin	Macrolides	24.5	19.7	1.2	7.5	-	-
Trimethoprim	Diaminopyrimidines	12	50	0.2	13	-	-
Trimethoprim_Sulfa[4]	Diaminopyrimidines	54	6	9.0	13	3	4.3
Tylosin	Macrolides	12.3	12.7	1.0	9.3	6.7	1.4
Virginiamycin	Streptogramins	-	-	-	2.6	2	1.3

All active substances were administered orally (through feed or drinking water). 1: lincomycin with spectinomycin. 2: spectinomycin with lincomycin. 3: sulfonamides with trimethoprim. 4: trimethoprim with sulfonamides. UDD/DDD$_{T}$ shows ratio with indication for treatment while UDD/DDD$_{P}$ shows ratio with indication for prevention scenario.

Questionnaire on antimicrobial usage

Confidentiality statement

Farmer: I declare that I was briefed about this study and I approve that all data collected on the farm will be used anonymously for further analysis and publications. The researchers can contact me or farm veterinarian within 1 month from the date of data collection, if data would be missing.

Date: ………………………… Name: …………………………

Signature: …………………………

Researcher: I declare that I have taken all necessary measures to inform the farmer about the purpose and context of this research and that the data collected on this farm will be processed anonymously and will only be used for further analysis and publication.

Date: ………………………… Name: …………………………

Signature: …………………………

Date of completion of questionnaire: ……………………………

Administrative details

Farm Identity (anonymous):

Farm name and address (for contact purpose only):

Contact number (farmer/ farm veterinarian):

Farm location:

Farm/ house characteristics

Indicate the sampling unit: House (one flock) Farm (all flocks)

1. Total number of poultry houses (having chicks) at farm ……………………………
2. How many chicks were actually set-up in the current round in the sampled house/ farm?
3. What was the average weight of the chicks at set-up?
4. How many chickens are currently present in the sampled house/ farm?
5. How old (in days) are the chickens currently present in the sampled house/ farm?
6. What is the actual weight of the chickens currently present in the sampled house/ farm?
7. (Expected) date of delivery to the slaughterhouse?

   Group 1: …………… Group 2: …………… Group 3: ……………
8. What is the actual feed and water intake per day?

Technical data

9. Average number of rounds / year?
10. Average number of chicks set up / round?

11. Number of chickens delivered to the slaughterhouse / year?
12. Average weight of chickens at slaughter?
13. Average duration of production cycle (in days) ?
14. Amount of feed used/purchased per year (kg)?
15. Is part of the feed produced on the farm itself?
16. Is there any feed being used at farm which is already mixed with antibiotics?

<u>On-farm antimicrobial use data</u>

Product name	Active substance(s)	Concentration	Unit	Used dose	Unit	Administration route	Number of birds treated	Treatment duration (days)	Actual Weight of birds at treatment (kg)	Age of birds at treatment (days)	Mian indication and purpose of use
